# Relationship Between Serum Myostatin and Endothelial Function in Non-Dialysis Patients with Chronic Kidney Disease

**DOI:** 10.3390/diseases12120328

**Published:** 2024-12-13

**Authors:** Ho-Hsiang Chang, Chih-Hsien Wang, Yu-Li Lin, Chiu-Huang Kuo, Hung-Hsiang Liou, Bang-Gee Hsu

**Affiliations:** 1 Division of Nephrology, Hualien Tzu Chi Hospital, Buddhist Tzu Chi Medical Foundation, Hualien 97004, Taiwan; 2 School of Medicine, Tzu Chi University, Hualien 97004, Taiwan; 3 Institute of Medical Sciences, Tzu Chi University, Hualien 97004, Taiwan; 4 School of Post-Baccalaureate Chinese Medicine, Tzu Chi University, Hualien 97004, Taiwan; 5 Division of Nephrology, Department of Internal Medicine, Hsin-Jen Hospital, New Taipei City 24243, Taiwan

**Keywords:** chronic kidney disease, digital thermal monitoring test, endothelial function, myostatin, vascular reactivity index

## Abstract

Background/Objectives: Myostatin, primarily produced by skeletal muscle, inhibits muscle growth and promotes protein degradation. It has been implicated in conditions such as obesity, insulin resistance, and cardiovascular disease. However, its association with endothelial function in chronic kidney disease (CKD) patients remains unclear. This study aimed to investigate the relationship between serum myostatin levels and endothelial function in 136 non-dialysis CKD patients at stages 3–5. Methods: Fasting blood samples were collected to measure serum myostatin levels using enzyme-linked immunosorbent assay kits. Endothelial function was evaluated non-invasively by measuring the vascular reactivity index (VRI) with a digital thermal monitoring test. Results: VRI values were classified as poor (<1.0, *n* = 25, 18.4%), intermediate (1.0 to <2.0, *n* = 63, 46.3%), or good (≥2.0, *n* = 48, 35.3%). Factors associated with poor vascular reactivity included older age (*p* = 0.026), elevated serum blood urea nitrogen (*p* = 0.020), serum creatinine (*p* = 0.021), urine protein-to-creatinine ratio (UPCR, *p* = 0.013), and myostatin levels (*p* = 0.003), along with reduced estimated glomerular filtration rate (*p* = 0.015). Multivariate regression analysis identified older age, higher serum creatinine, and log-transformed myostatin levels as significant independent predictors of lower VRI. Conclusions: These findings suggest that myostatin may serve as a potential biomarker for endothelial dysfunction in CKD patients. Future large-scale, longitudinal studies are warranted to confirm and extend our preliminary findings.

## 1. Introduction

Chronic kidney disease (CKD) affects an estimated 850 million people worldwide [1] and is associated with a marked increase in cardiovascular (CV) morbidity and mortality. This heightened risk extends beyond traditional factors such as hypertension (HTN), diabetes mellitus (DM), and dyslipidemia [2]. Research has identified both macrovascular and microvascular endothelial dysfunction as independent predictors of CV events, particularly in individuals at risk of coronary artery disease [3].

Endothelial dysfunction offers valuable insights into CV risk that extend beyond conventional risk factors [4]. Studies have confirmed the presence of endothelial dysfunction in both large and small arteries of CKD patients [5], with its severity closely correlating with the extent of renal damage [6].

Endothelial dysfunction is a key nontraditional risk factor for CV disease in CKD patients [7]. The vascular endothelium plays a vital role in maintaining vascular health, facilitating the regulated exchange of fluids, molecules, and cells, and producing nitric oxide (NO), which is essential for vasodilation. Patients with CKD experience significant impairments in endothelium-dependent vasodilation, which worsen with CKD progression and are independently linked to adverse CV outcomes [8]. The development of endothelial dysfunction in CKD is driven by several factors, including oxidative stress, advanced glycation end products, inflammation, low vitamin D levels, hyperphosphatemia, elevated fibroblast growth factor 23, reduced α-Klotho, and the accumulation of endothelial nitric oxide synthase (eNOS) inhibitors [9,10]. Together, these factors reduce NO bioavailability, impairing vascular function in CKD patients.

Myostatin is a member of the transforming growth factor-β (TGF-β) superfamily and is primarily expressed in skeletal muscle, where its production and secretion are regulated by factors such as oxidative stress, hyperammonemia, angiotensin II signaling, inflammatory mediators, and glucocorticoids [11]. Elevated myostatin levels have been observed during processes such as vascular smooth muscle cell (VSMC) activation, the chemotaxis of monocytes, and the remodeling of the vascular wall [11]. Emerging research suggests that myostatin contributes to vascular damage through direct or indirect interactions with activin or TGF-β signaling pathways [12]. In aortic endothelial cells, myostatin exposure activates TGF-β signaling, decreases the phosphorylation of eNOS, and increases the levels of pro-atherogenic adhesion molecules [13], indicating that vessel wall components are direct targets of myostatin. Additionally, myostatin has been detected at various stages of atherosclerosis in the human aorta [14]. Further studies highlight that myostatin expression is strongly induced by indoxyl sulfate, a uremic toxin [15]. Indoxyl sulfate influences VSMC phenotype changes, including inflammation and proliferation, which are closely associated with atherosclerosis in uremic vasculopathy [16].

The role of myostatin in endothelial dysfunction, a major factor in cardiovascular disease among patients with chronic kidney disease (CKD), is not well understood. Specifically, the potential of circulating myostatin to serve as a standalone indicator of endothelial dysfunction in CKD patients who are not on dialysis has yet to be fully established. This study aimed to examine the relationship between myostatin levels and the vascular reactivity index (VRI)—a reliable and non-invasive indicator of endothelial function assessed using temperature-based digital monitoring—in patients with CKD stages 3–5 who are not receiving dialysis.

## 2. Materials and Methods

### 2.1. Participants

From October 2020 to March 2021, 136 individuals with chronic kidney disease (CKD) stages 3–5 not requiring dialysis were enrolled at Hualien Tzu Chi Medical Center. The research protocol was approved by the Hualien Tzu Chi Hospital Ethics Committee (IRB 108-219-A), and all participants signed informed consent forms. Adults aged 20 and older with stage 3–5 CKD, characterized by an estimated glomerular filtration rate (eGFR) below 60 mL/min sustained for at least three months, were eligible for inclusion. Exclusion criteria included conditions such as recent acute myocardial infarction, chronic obstructive pulmonary disease, amputations, liver cirrhosis, heart failure, acute infections, and malignancies.

Blood pressure (BP) was assessed using a traditional mercury sphygmomanometer following a 10 min period of rest. Systolic BP (SBP) and diastolic BP (DBP) readings were determined by identifying the Korotkoff sounds during the measurement process. Three BP readings were obtained at 5 min intervals, and the mean of these readings was used for analysis. Hypertension (HTN) was classified as an average BP of ≥140/90 mmHg or the use of medications for blood pressure control. Diabetes mellitus (DM) was diagnosed if fasting plasma glucose was ≥126 mg/dL, 2 h post-oral glucose tolerance test levels were ≥200 mg/dL, or if hypoglycemic drugs or insulin were prescribed. Chronic glomerulonephritis (GN) was identified through the electronic record. Smoking status was collected, with individuals categorized as smokers if they had ever smoked or were current smokers. Data on medication use, including angiotensin receptor blockers (ARBs), β-blockers, calcium channel blockers (CCBs), statins, and fibrates, was also collected.

### 2.2. Anthropometric Analyses

Body weight was recorded to the nearest 0.5 kg, and participants were dressed in light clothing and without shoes. Height was measured to the nearest 0.5 cm. Body mass index (BMI) was determined by dividing weight in kilograms by the square of height in meters (kg/m^2^). Body composition, including lean body mass (kg) and fat mass (kg), was measured among 104 participants using a tetrapolar bioelectrical impedance device 90 (Biodynamics^®^ BIA 450 Bioimpedance Analyzer, Seattle, WA, USA).

### 2.3. Biochemical Investigations and CKD Stage

A fasting blood sample of about 5 mL was collected and centrifuged at 3000× *g* for 10 min to separate the serum. Biochemical parameters, including total cholesterol (TCH), triglycerides (TG), low-density lipoprotein cholesterol (LDL-C), fasting glucose, albumin, blood urea nitrogen (BUN), creatinine, calcium, and phosphorus, were measured using an automated analyzer (Siemens Advia 1800, Siemens Healthcare GmbH, Erlangen, Germany). High-density lipoprotein cholesterol (HDL-C) was calculated using the formula HDL-C = TCH − LDL-C − (TG/5) [17]. Serum myostatin levels were quantified using a commercially available enzyme-linked immunosorbent assay kit from R&D Systems, Inc. (Minneapolis, MN, USA) [18]. A random urine sample was also analyzed to determine the urine protein-to-creatinine ratio (UPCR).

The eGFR was calculated as the average of results from tests performed at intervals of at least three months, using the Chronic Kidney Disease Epidemiology Collaboration (CKD-EPI) equation. Based on the Kidney Disease Outcomes Quality Initiative (KDOQI) recommendations, participants were classified into stages of chronic kidney disease (CKD): stage 3 (eGFR 30–59 mL/min/1.73 m^2^), stage 4 (eGFR 15–29 mL/min/1.73 m^2^), and stage 5 (eGFR below 15 mL/min/1.73 m^2^).

### 2.4. Endothelial Function Measurements

After a night of fasting and avoiding caffeine, tobacco, alcohol, and any vasoactive medications, endothelial function was evaluated using the VENDYS-II, a digital thermal monitoring device approved by the FDA (Endothelix Inc., Houston, TX, USA) [19]. Participants rested supine at room temperature (22–24 °C) for 30 min before testing. Blood pressure cuffs were fitted around the upper arms, while temperature sensors were positioned on the index fingers. Measurements were recorded from both hands during three phases: a 5 min stabilization period, a 5 min cuff inflation (50 mmHg above SBP), and a 5 min deflation. As the cuff was released, the swift flow of blood into the forearm and hand caused a noticeable increase in fingertip temperature, reflecting the reactive hyperemia response. VENDYS software analyzed this effect by assessing the peak variation between the temperature rebound curve and the baseline zero-reactivity curve during the hyperemic phase, resulting in the calculation of VRI. Based on the VRI values, participants were classified into three categories of vascular reactivity: good (≥2.0), intermediate (1.0 to <2.0), and poor (<1.0) [20].

### 2.5. Statistical Analyses

To detect a correlation coefficient of approximately 0.25 between serum myostatin levels and VRI, with an alpha level of 0.05 and a power of 80%, a minimum of 123 patients should be included in the study.

Data were tested for normality with the Kolmogorov–Smirnov test. Normally distributed data were presented as mean ± standard deviation and compared using a two-tailed Student’s *t*-test. For non-normally distributed data, comparisons between patient groups were performed using the Kruskal–Wallis test or one-way analysis of variance (ANOVA), depending on the distribution of the variables. Categorical variables were summarized as counts along with their corresponding percentages and analyzed using a chi-square test. Both univariate and multivariate logistic regression analyses were conducted to assess the relationships between myostatin levels and vascular reactivity dysfunction (categorized as intermediate/poor) or poor vascular reactivity. Additionally, a receiver operating characteristic (ROC) curve analysis was utilized to evaluate the effectiveness of myostatin levels in predicting vascular reactivity dysfunction or poor vascular reactivity. Parameters with non-normal distribution (i.e., TCH, TG, fasting glucose, albumin, eGFR, UPCR, and myostatin levels) were logarithmically transformed (log-) before further analysis. Univariate linear regression analysis assessed the relationship between the variables and VRI values. Variables that yielded significant findings (*p* < 0.05) were then incorporated into a multivariate stepwise linear regression analysis for further examination. The relationships between log-myostatin levels and clinical variables were examined using Spearman’s rank correlation coefficient analysis. Statistical analyses were performed using SPSS version 19.0 software (IBM Corp., Armonk, NY, USA), with a *p*-value less than 0.05 considered to indicate statistical significance.

## 3. Results

The clinical characteristics of the study population are summarized in Table 1. Among the 136 participants, 48 (35.3%) were women, 71 (52.2%) had DM, and 112 (82.4%) had HTN. Fifteen of our participants (11%) were classified as smokers. Within this CKD cohort, 25 (18.4%), 63 (46.3%), and 48 (35.3%) were classified as having poor, intermediate, and good VRI, respectively.

As VRI decreased, significant increases were observed in age (*p* = 0.026), myostatin levels (*p* = 0.003), serum creatinine (*p* = 0.021), BUN (*p* = 0.020), and UPCR (*p* = 0.013), while eGFR significantly decreased (*p* = 0.015). No significant differences were found across the three VRI groups in terms of sex, smoking, DM, HTN, BMI, lean body and fat mass, or the use of anti-HTN drugs, statins, or fibrates.

After controlling for variables such as gender, smoking status, age, BMI, blood pressure, eGFR, UPCR, albumin, fasting glucose levels, lipid profile (TCH and TG), phosphorus, calcium, and serum myostatin, logistic regression analysis revealed an independent association between serum myostatin and impaired vascular reactivity. Specifically, serum myostatin was significantly correlated with vascular reactivity dysfunction (odds ratio [OR] 1.438, 95% confidence interval [CI] 1.057–1.955; *p* = 0.021) and reduced vascular responsiveness (OR 1.576, 95% CI 1.189–2.089; *p* = 0.002) in individuals with CKD (Table 2).

**Figure 1:**
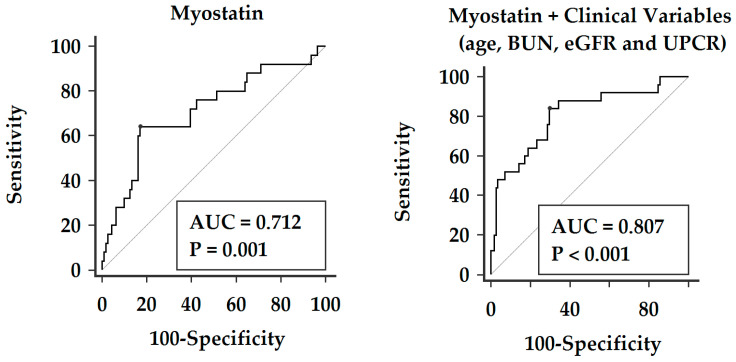
ROC curves for myostatin alone and with added clinical variables in predicting poor vascular reactivity. Age, BUN, eGFR, and UPCR were included in the model due to their significant differences across vascular reactivity index groups.

ROC curve analysis indicated that myostatin could only modestly predict vascular reactivity dysfunction (area under the curve [AUC] 0.605, 95% CI 0.508–0.702; *p* = 0.034) and moderately predict poor vascular reactivity (AUC 0.712, 95% CI 0.590–0.835; *p* = 0.0007) (Table 3). By adding clinical variables with significant differences across VRI groups—including age, BUN, eGFR, and UPCR—the AUC increased to 0.807 (95% CI 0.731–0.870; *p* < 0.0001) (Figure 1). The predicted formula is provided in Supplementary Table S1.

Table 4 presents the correlation between clinical characteristics and serum VRI. Univariate linear regression analysis showed that VRI was positively correlated with log-eGFR (*r* = 0.271, *p* = 0.001) but negatively correlated with advanced age (*r* = −0.248, *p* = 0.004); serum log-BUN (*r* = −0.262, *p* = 0.002); log-creatinine (*r* = −0.277, *p* = 0.001); log-UPCR (*r* = −0.244, *p* = 0.004); and log-myostatin (*r* = −0.263, *p* = 0.002). Multivariate forward stepwise linear regression analysis demonstrated that VRI had an independent association with elevated serum log-myostatin (β = −0.256, R^2^ change = 0.057, *p* = 0.002), older age (β = −0.331, R^2^ change = 0.081, *p* < 0.001), and log-creatinine levels (β = −0.273, R^2^ change = 0.070, *p* = 0.001).

Spearman’s rank correlation coefficient analysis revealed that serum log-myostatin level had a positive correlation with log-UPCR (*r* = 0.230, *p* = 0.007) and lean body mass (*r* = 0.243, *p* = 0.013) and negatively correlated with serum creatinine level (*r* = −0.217, *p* = 0.011) and VRI (*r* = −0.263, *p* = 0.002) (Table 5).

## 4. Discussion

This cross-sectional study of non-dialysis CKD stage 3–5 patients found that a decline in eGFR, older age, elevated UPCR, and increased myostatin levels were linked to poor vascular reactivity. Higher myostatin levels also posed an independent risk for vascular dysfunction, regardless of other variables. These results suggested that myostatin may be used as a biomarker of endothelial dysfunction in a non-dialysis CKD stage 3–5 population, although this warrants future mechanistic studies. Previous research has demonstrated that worsening endothelial dysfunction with eGFR deterioration and increased myostatin levels in early atherosclerotic lesions contributed to uremic vasculopathy [16]. Our findings provided additional support for this association.

Endothelial dysfunction, including atherosclerosis, hemostatic disruption, and inflammation, is prevalent among CKD patients and plays a key role in CV outcomes like coronary artery and cerebrovascular diseases [4,5,21,22]. The use of non-invasive methods or biomarkers to evaluate endothelial function and assess adverse outcomes in patients with CKD is gaining attention. The VRI is a non-invasive measure and non-operator-dependent tool of endothelial function that correlates with atherosclerosis risk factors and coronary artery disease in a large registry database including 6084 patients [20]. In patients with CKD, the vascular reactivity is predictive of CV [23] and cerebrovascular events [24].

Dysregulated endothelium-dependent vascular relaxation is highly correlated with aging in the renal and coronary arteries. Moreover, it has been discovered that endothelial dysfunction, a factor in the formation of atherosclerosis, raises the risk of CV illnesses [25]. Therefore, endothelial dysfunction is a key mechanism of increased risk of CV disease in older individuals [26]. The association between progressive renal impairment and reduced endothelium-dependent vasodilation has been established in patients with CKD [27]. Animal and human studies have demonstrated that CKD leads to endothelial dysfunction through increased endothelial permeability, endothelium-dependent vasodilation, and elevated proinflammatory cytokine levels [8,9,10,28]. Another study demonstrated a strong association between endothelial dysfunction, inflammatory activity, and increased urinary albumin excretion over a 10-year follow-up [29].

The positive association between myostatin and endothelial dysfunction in CKD highlighted the mechanisms of CKD-associated vasculopathy. In vitro studies have shown that VSMCs upregulate C-C motif chemokine receptor 2 (CCR2) and monocyte chemoattractant protein 1 (MCP1) following exposure to myostatin. MCP1 is a key mediator of vascular inflammation and accelerates atherogenesis. In addition, MCP1-exposed cells produce myostatin, suggesting that myostatin propagates vascular inflammation. Additionally, myostatin hampers the cell cycle progression of VSMCs by extending the G0/G1 phase and lowering their proliferation rate. Exposure to myostatin disrupts the cytoskeletal structure and promotes the migration of VSMCs from the media layer to the intima layer of the arteries, a key mechanism contributing to the formation of atherosclerosis [14]. Research on muscle biopsy samples from patients with advanced CKD showed that myostatin expression was linked to muscle sarcolemma. This suggests that myostatin is produced locally within the muscle, released into the bloodstream, and functions as an endocrine cytokine [30]. Increased myostatin production and decreased renal excretion were postulated to increase myostatin level in the early stages of CKD and further increase as the eGFR declines [31]. Myostatin expression increases during early vascular wall activation and reaches higher levels in progressive atherosclerotic lesions. Myostatin is closely linked to the activation of VSMCs, monocyte chemotaxis, and vascular wall remodeling [14]. Furthermore, VSMC myostatin, when stimulated by uremic toxins, can accelerate atherosclerosis and contribute to vascular cell aging in patients with CKD [32].

Myostatin is a myokine produced by skeletal muscle. The positive correlation between serum myostatin levels and lean body mass observed in our study is consistent with previous reports in CKD [33].

Our study found an inverse association between serum myostatin and eGFR, which may contribute to impaired renal clearance, enhanced inflammation status, and endothelial dysfunction in advanced CKD [34]. Similarly, in another CKD cohort, serum myostatin was inversely correlated with eGFR, presumably due to renal underexcretion [35].

There were certain restrictions in this study. First, the sample size of the CKD population was limited by the single-center design; larger sample numbers are needed to confirm the results of this study. Second, previous research has shown that age, sex, blood pressure, and smoking are associated with endothelial dysfunction [15,36,37]. The absence of associations between vascular reactivity and BP and sex was likely attributable to the high frequency of comorbidities in the study population, while the association between smoking and endothelial dysfunction may be hampered due to the small number of smoking cases. Third, alcohol consumption history was not collected in our study, but may impact CV function. Fourth, the AUC value of 0.712 for myostatin alone indicates only moderate predictive value, suggesting that other biomarkers with greater predictive ability, or a combination of biomarkers, would be necessary to enhance clinical utility for prediction. Fifth, lean body mass and fat mass were measured in 76.5% of our patients. Sixth, one notable limitation of our study is the absence of apolipoprotein measurements, such as Apo A-I and Apo B, which are critical components of lipid metabolism and have been increasingly recognized as significant markers for cardiovascular risk. For example, the Apo B/Apo A-I ratio provides insights into the balance between atherogenic and anti-atherogenic lipoproteins [38], offering a more nuanced understanding of lipid-related CV risk than traditional cholesterol profiles. While our study focused on traditional lipid markers, incorporating apolipoproteins in future research could enhance the characterization of cardiovascular risk and deepen our understanding of the interplay between lipid metabolism, myostatin levels, and endothelial dysfunction in CKD populations. Finally, given the cross-sectional design of our study, our findings should be regarded as hypothesis-generating, and causal relationships between myostatin levels and endothelial dysfunction cannot be established.

## 5. Conclusions

Our study suggests that serum myostatin may serve as an independent predictor of endothelial dysfunction in non-dialysis CKD stages 3–5. However, combining it with other relevant clinical variables or biomarkers is warranted to enhance its predictive ability for endothelial dysfunction. Furthermore, our preliminary findings encourage future large-scale, longitudinal studies to confirm causality and investigate its utility in forecasting adverse CV outcomes.

## Figures and Tables

**Table 1:** Clinical characteristics by vascular reactivity index in chronic kidney disease patients.

Characteristics	All Patients (*n* = 136)	Good Vascular Reactivity (*n* = 48)	Intermediate Vascular Reactivity (*n* = 63)	Poor Vascular Reactivity (*n* = 25)	*p*-Value
Age (years)	66.06 ± 11.77	63.60 ± 8.89	65.83 ± 13.83	71.36 ± 9.44	0.026 *
Male, *n* (%)	88 (64.7)	31 (64.6)	41 (65.1)	16 (64.0)	0.995
CKD etiologies					
Diabetes mellitus, *n* (%)	71 (52.2)	24 (50.0)	36 (57.1)	11 (44.0)	0.501
Chronic GN, *n* (%)	45 (33.1)	16 (33.3)	19 (30.2)	10 (40.0)	0.675
Height (cm)	161.22 ± 8.83	160.38 ± 8.66	161.03 ± 8.82	161.91 ± 9.06	0.762
Body weight (kg)	69.93 ± 16.96	71.14 ± 16.20	69.71 ± 14.33	68.18 ± 23.83	0.773
Body mass index (kg/m^2^)	26.49 ± 4.47	26.97 ± 4.58	26.74 ± 4.53	24.95 ± 3.90	0.157
Lean body mass (kg) ^a^	50.83 ± 11.27	50.81 ± 12.37	51.71 ± 9.70	47.85 ± 13.45	0.511
Fat mass (kg) ^a^	17.89 ± 7.19	18.90 ± 7.07	17.59 ± 7.60	16.36 ± 5.99	0.471
Vascular reactivity index	1.65 ± 0.74	2.42 ± 0.34	1.50 ± 0.32	0.54 ± 0.22	<0.001 *
Systolic blood pressure (mmHg)	144.81 ± 26.95	138.51 ± 27.76	148.37 ± 22.65	147.96 ± 33.56	0.130
Diastolic blood pressure (mmHg)	82.10 ± 12.01	79.48 ± 11.28	82.63 ± 11.42	85.76 ± 14.01	0.093
Total cholesterol (mg/dL)	150.00 (134.25–175.00)	150.00 (136.25–181.00)	152.00 (126.00–174.00)	148.00 (134.50–170.50)	0.988
Triglyceride (mg/dL)	110.50 (99.00–164.50)	110.00 (86.00–191.75)	109.00 (78.00–161.00)	112.00 (71.50–151.00)	0.740
LDL-C (mg/dL)	82.68 ± 35.94	80.50 ± 30.35	82.63 ± 30.00	86.96 ± 55.75	0.769
HDL-C (mg/dL)	46.50 ± 21.12	45.02 ± 19.63	44.43 ± 19.05	54.54 ± 27.11	0.107
Fasting glucose (mg/dL)	102.50 (93.00–129.25)	100.50 (94.00–128.00)	101.00 (90.00–127.00)	110.00 (98.00–140.50)	0.448
Albumin (g/dL)	4.20 (4.00–4.40)	4.30 (4.20–4.40)	4.20 (4.00–4.40)	4.10 (3.60–4.35)	0.084
Blood urea nitrogen (mg/dL)	52.32 ± 24.03	44.75 ± 24.14	55.49 ± 24.47	58.88 ± 19.36	0.020 *
Creatinine (mg/dL)	3.48 ± 1.88	2.91 ± 1.48	3.68 ± 1.99	4.08 ± 2.07	0.021 *
eGFR (mL/min)	18.11 (12.76–35.57)	23.81 (14.82–46.30)	17.43 (12.52–31.70)	14.66 (11.95–26.60)	0.015 *
UPCR (mg/g)	858.90 (306.26–2369.29)	435.06 (180.46–2259.72)	873.20 (356.40–2364.24)	1992.09 (803.80–3440.55)	0.013 *
Total calcium (mg/dL)	8.72 ± 0.53	8.71 ± 0.57	8.72 ± 0.44	8.73 ± 0.67	0.975
Phosphorus (mg/dL)	4.25 ± 1.02	4.12 ± 0.81	4.19 ± 0.95	4.66 ± 1.42	0.078
Myostatin (ng/mL)	2.84 (1.48–3.60)	2.63 (1.24–3.29)	2.66 (1.51–3.61)	3.67 (2.79–5.04)	0.003 *
Myostatin—male (ng/mL)	2.99 (1.89–3.78)	2.75 (1.31–3.27)	2.77 (1.83–3.91)	3.75 (3.18–6.83)	0.003 *
Myostatin—female (ng/mL)	2.61 (0.96–3.37)	2.04 (0.73–3.35)	2.32 (0.97–3.15)	3.06 (1.66–3.99)	0.412
Smoker, *n* (%)	15 (11.0)	8 (16.7)	6 (9.5)	1 (4.0)	0.228
Hypertension, *n* (%)	112 (82.4)	40 (83.3)	50 (79.4)	22 (88.0)	0.617
ARB use, *n* (%)	69 (50.7)	22 (45.8)	31 (49.2)	16 (64.0)	0.320
β-blocker use, *n* (%)	55 (40.4)	16 (33.3)	29 (46.0)	10 (40.0)	0.401
CCB use, *n* (%)	61 (44.9)	20 (41.7)	28 (44.4)	13 (52.0)	0.699
Statin use, *n* (%)	65 (47.8)	20 (41.7)	30 (47.6)	15 (60.0)	0.330
Fibrate use, *n* (%)	30 (22.1)	10 (20.8)	17 (27.0)	3 (12.0)	0.301
CKD stage 3, *n* (%)	45 (33.1)	22 (45.8)	18 (28.6)	5 (20.0)	0.059
CKD stage 4, *n* (%)	36 (26.5)	14 (29.2)	16 (25.4)	6 (24.0)	
CKD stage 5, *n* (%)	55 (40.4)	12 (25.0)	29 (46.0)	14 (56.0)	

Continuous variables are expressed as mean ± standard deviation and were assessed with one-way analysis of variance. For non-normally distributed data, median and interquartile range values are reported and were analyzed using the Kruskal–Wallis test. Categorical variables are presented as counts and percentages, with comparisons made using the chi-square test. The abbreviations used in this study are as follows: CKD (chronic kidney disease), GN (glomerulonephritis), LDL-C (low-density lipoprotein cholesterol), HDL-C (high-density lipoprotein cholesterol), eGFR (estimated glomerular filtration rate), UPCR (urine protein-to-creatinine ratio), ARB (angiotensin receptor blocker), and CCB (calcium channel blocker). * Statistical significance was defined as a *p*-value below 0.05. ^a^ In total, 104 (76.5%) patients had lean body mass and fat mass measurements.

**Table 2:** Multivariate logistic regression analysis of myostatin for vascular reactivity dysfunction or poor vascular reactivity.

Model	Myostatin for Vascular Reactivity Dysfunction		Myostatin for Poor Vascular Reactivity	
	OR (95% CI)	*p*-Value	OR (95% CI)	*p*-Value
Crude model	1.372 (1.074–1.752)	0.011 *	1.436 (1.144–1.802)	0.002 *
Adjusted model	1.438 (1.057–1.955)	0.021 *	1.576 (1.189–2.089)	0.002 *

The adjusted model accounted for variables including sex, age, smoking, body mass index, systolic and diastolic blood pressure, estimated glomerular filtration rate, urine protein-to-creatinine ratio, fasting glucose, albumin, total cholesterol, triglycerides, total calcium, phosphorus, and myostatin. OR, odds ratio. CI, confidence interval. * A *p*-value of less than 0.05 was deemed statistically significant.

**Table 3:** The diagnostic relevance of myostatin for identifying vascular reactivity dysfunction or impaired vascular reactivity was assessed.

	**Vascular Reactivity Dysfunction**						
	**AUC (95% CI)**	***p*-Value**	**Cut-Off**	**Sensitivity (%)**	**Specificity (%)**	**PPV (%)**	**NPV (%)**
**Myostatin (ng/mL)**	0.605 (0.508–0.702)	0.034 *	3.57	36.7	93.8	91.4	44.6
	**Poor vascular reactivity**						
	**AUC (95% CI)**	***p*-Value**	**Cut-off**	**Sensitivity (%)**	**Specificity (%)**	**PPV (%)**	**NPV (%)**
**Myostatin (ng/mL)**	0.712 (0.590–0.835)	0.0007 *	3.57	64.0	82.9	45.7	91.1

AUC, area under the curve; 95% CI, 95% confidence interval; PPV, positive predictive value; NPV, negative predictive value. * A *p*-value of less than 0.05 was regarded as statistically significant.

**Table 4:** The vascular reactivity index and various clinical variables were examined using simple or multivariate linear analyses.

Variables	Vascular Reactivity Index				
	Simple Regression		Multivariate Regression		
	*r*	*p*-Value	Beta	Adjusted R^2^ Change	*p*-Value
Male	−0.007	0.939			
Female	0.007	0.939	–	–	–
Diabetes mellitus	−0.013	0.878	–	–	–
Hypertension	−0010	0.912	–	–	–
Age (years)	−0.248	0.004 *	−0.331	0.081	<0.001 *
Height (cm)	0.007	0.935	–	–	–
Body weight (kg)	0.127	0.140	–	–	–
Body mass index (kg/m^2^)	0.159	0.064	–	–	–
Lean body mass (kg) ^a^	0.029	0.774	–	–	–
Fat mass (kg) ^a^	0.131	0.185	–	–	–
Systolic blood pressure (mmHg)	−0.082	0.342	–	–	–
Diastolic blood pressure (mmHg)	−0.157	0.068	–	–	–
Log-TCH (mg/dL)	−0.023	0.788	–	–	–
Log-triglyceride (mg/dL)	0.065	0.453	–	–	–
LDL-C (mg/dL)	−0.045	0.605	–	–	–
HDL-C (mg/dL)	−0.107	0.215			
Log-glucose (mg/dL)	−0.087	0.312	–	–	–
Log-albumin (g/dL)	0.137	0.111	–	–	–
BUN (mg/dL)	−0.262	0.002 *	–	–	–
Creatinine (mg/dL)	−0.277	0.001 *	−0.273	0.070	0.001 *
Log-eGFR (mL/min)	0.271	0.001 *	–	–	–
Log-UPCR (mg/g)	−0.244	0.004 *	–	–	–
Total calcium (mg/dL)	−0.027	0.758	–	–	–
Phosphorus (mg/dL)	−0.152	0.077	–	–	–
Log-myostatin (ng/mL)	−0.263	0.002 *	−0.256	0.057	0.002 *

Log transformation was applied before analysis because the data for total cholesterol, triglycerides, fasting glucose, albumin, eGFR, UPCR, and myostatin showed a skewed distribution. Simple linear regression and multivariate stepwise linear regression were conducted, with factors such as age, blood urea nitrogen (BUN), creatinine, log-eGFR, log-UPCR, and log-myostatin included in the models. Abbreviations used are BUN (blood urea nitrogen), TCH (total cholesterol), LDL-C (low-density lipoprotein cholesterol), HDL-C (high-density lipoprotein cholesterol), eGFR (estimated glomerular filtration rate), and UPCR (urine protein-to-creatinine ratio). * A *p*-value of less than 0.05 was considered statistically significant. ^a^ In total, 104 (76.5%) patients had lean body mass and fat mass measurements.

**Table 5:** Spearman’s rank correlation coefficient analysis between clinical factors and log-transformed myostatin level.

Variables	Spearman’s Correlation Coefficient	*p*-Value
Age (years)	−0.158	0.067
Body mass index (kg/m^2^)	0.066	0.446
Lean body mass (kg) ^a^	0.243	0.013 *
Fat mass (kg) ^a^	0.062	0.533
Vascular reactivity index	−0.263	0.002 *
Systolic blood pressure (mmHg)	0.027	0.757
Diastolic blood pressure (mmHg)	0.151	0.079
Log-TCH (mg/dL)	0.062	0.472
Log-triglyceride (mg/dL)	−0.014	0.870
LDL-C (mg/dL)	0.067	0.439
HDL-C (mg/dL)	0.054	0.533
Log-glucose (mg/dL)	−0.147	0.087
Log-albumin (g/dL)	−0.090	0.300
Blood urea nitrogen (mg/dL)	0.094	0.461
Creatinine (mg/dL)	−0.217	0.011 *
Log-eGFR (mL/min)	−0.156	0.069
Log-UPCR (mg/g)	0.230	0.007 *
Total calcium (mg/dL)	0.046	0.592
Phosphorus (mg/dL)	0.109	0.206

The distributions of fasting glucose, total cholesterol (TCH), triglycerides, albumin, UPCR, eGFR, and myostatin were non-normal; thus, these variables were log-transformed prior to statistical analysis. Abbreviations include TCH (total cholesterol), LDL-C (low-density lipoprotein cholesterol), HDL-C (high-density lipoprotein cholesterol), UPCR (urinary protein-to-creatinine ratio), and eGFR (estimated glomerular filtration rate). * Statistical significance was defined as a *p*-value below 0.05. ^a^ In total, 104 (76.5%) patients had lean body mass and fat mass measurements.

## Data Availability

The raw data supporting the conclusions of this article will be made available by the authors on request.

## Supplementary Materials

The following supporting information can be downloaded at https://www.mdpi.com/article/10.3390/diseases12120328/s1: Table S1: The equation for myostatin clinical variables in the receiver operating characteristic (ROC) curve analysis.
