# Supplementary material for: Relationship Between Serum Myostatin and Endothelial Function in Non-Dialysis Patients with Chronic Kidney Disease
Source: Diseases. 2024 Dec 13;12(12):328. doi: 10.3390/diseases12120328 (PMC11726979; doi:10.3390/diseases12120328)
Supplement: Supplementary file 1 [file diseases-12-00328-s001.zip › diseases-3347263-supplementary.pdf]

Table S1: The equation for myostatin clinical variables in the receiver operating characteristic (ROC) curve analysis.

Using a multivariate logistic regression model with independent variables (log-myostatin, age, BUN, eGFR, and UPCR), the probability of poor vascular reactivity was represented by the equation below:

Probability (Poor VRI) =

$$\frac{e^{(\beta_0+\beta_1 \text{log-myostatin}+\beta_2 \text{age}+\beta_3 \text{BUN}+\beta_4 \text{eGFR}+\beta_5 \text{UPCR})}}{1+e^{(\beta_0+\beta_1 \text{log-myostatin}+\beta_2 \text{age}+\beta_3 \text{BUN}+\beta_4 \text{eGFR}+\beta_5 \text{UPCR})}}$$

β0 is the intercept

β1 to β5 are regression coefficients representing the strength of association between each variable and the outcome

β0 = -13.279

β1 = 3.122

β2 = 0.088

β3 = 0.014

β4 = 0.018

β5 = 1.037
